# A synergistic multimodality treatment approach to address the key drivers of wound chronicity

**DOI:** 10.1016/j.jvsv.2025.102348

**Published:** 2025-10-23

**Authors:** Joann M. Lohr, Joseph D. Raffetto, David J. Dexter, Matthew J. Regulski, Michael E. Edmonds, Kathleen J. Ozsvath, Melodie M. Blakely

**Affiliations:** a Department of Surgery, William Jennings Bryan Dorn VA Medical Center, Columbia, SC; b Section of Vascular Surgery, VA Boston Healthcare System, Boston, MA; c Department of Surgery, Brigham and Women's Hospital, Boston, MA; d Department of Vascular Surgery, Sentara Vascular Specialists, Norfolk, VA; e Division of Vascular Surgery, Eastern Virginia Medical School, Norfolk, VA; f Department of Foot and Ankle Surgery, Ocean County Foot and Ankle Surgical Associates, PC, Forked River, NJ; g Department of Diabetes, School of Cardiovascular Medicine and Metabolic Sciences, King’s College London, London, United Kingdom; h Department of Vascular Surgery, Vascular Associates, St Peters Health Partners, Troy, NY; i Department of Market Access, Real World Evidence, AOTI, Inc, Oceanside, CA

**Keywords:** Advanced wound care, Chronic wound, Intermittent compression (IC), Topical oxygen therapy (TOT), Wound pathophysiology, Intermittent topical oxygen therapy (ITOT)

## Abstract

**Background:**

Chronic wounds remain a major clinical and economic burden, affecting millions worldwide. Despite advances in wound care, many wounds fail to heal due to persistent tissue hypoxia, unresolved inflammation, lymphatic dysfunction, edema, and ischemia-reperfusion injury. These interrelated mechanisms are further compounded by comorbidities such as obesity, diabetes, and vascular disease, highlighting the need for therapeutic approaches that address multiple barriers to repair simultaneously.

**Methods:**

We review the pathophysiological drivers of wound chronicity—including the inflammation/edema/hypoxia cycle, endothelial dysfunction, and impaired lymphatic clearance—and summarize evidence on the roles of oxygen, nitric oxide, redox signaling, mechanotransduction, and specialized pro-resolving lipid mediators in tissue repair. We then evaluate two complementary, noninvasive interventions: topical oxygen therapy, which directly elevates wound tissue oxygen tension to support oxidative burst, angiogenesis, collagen synthesis, and specialized pro-resolving lipid mediator biosynthesis; and intermittent compression, which enhances lymphatic drainage, reduces edema, normalizes capillary gradients, and activates mechanosensitive repair pathways in endothelial cells, macrophages, fibroblasts, and keratinocytes.

**Results:**

Chronic wound pathophysiology involves overlapping mechanisms of hypoxia, inflammation, edema, endothelial dysfunction, and reperfusion injury. Both topical oxygen therapy and intermittent compression independently improve oxygen delivery, perfusion, inflammation resolution, and tissue remodeling. When combined as intermittent topical oxygen therapy (ITOT), these modalities exert synergistic effects, amplifying oxygen bioavailability and potentiating anti-inflammatory, angiogenic, and reparative signaling. Clinical studies demonstrate that ITOT significantly increases healing rates, reduces healing time, lowers recurrence, and decreases hospitalizations and amputations in chronic wounds. Cost-effectiveness analyses further indicate improved quality-adjusted life years and reduced long-term expenditures.

**Conclusions:**

Chronic wounds persist due to a self-sustaining cycle of hypoxia, edema, and inflammation. By integrating oxygen delivery with cyclical compression, ITOT directly addresses the multifactorial barriers to repair, promoting durable healing and reducing complications. This multi-modality approach represents a promising therapeutic advance in the management of refractory lower extremity wounds, with broad implications for improving outcomes and quality of life and reducing health care costs.

**Article Highlights**

• **Type of Article:** Comprehensive review of mechanistic, translational, and clinical evidence, including randomized controlled trials and cohort studies·
• **Key Findings:** Combining topical oxygen therapy and intermittent compression addresses hypoxia, inflammation, and lymphatic dysfunction in chronic wounds. Clinical studies show intermittent topical oxygen therapy increases healing rates (up to 56% at 12 months), reduces recurrence (6.7% vs 40%), hospitalizations (7.1% vs 40%), and amputations (8.6% vs 31.4%) compared with standard care.
• **Take Home Message:** A synergistic, multimodality approach integrating topical oxygen and intermittent compression directly targets the core drivers of wound chronicity, resulting in improved healing, fewer complications, and reduced health care utilization in refractory lower extremity wounds.

Chronic wounds represent a significant clinical and economic burden, affecting millions of patients worldwide and contributing to substantial health care costs. Despite advances in wound care, many wounds remain refractory.^1^ There is a pressing need for innovative therapeutic solutions that wholistically address critical barriers to healing and optimize healed tissue quality, durability, and function.^2^

## Why wounds become chronic: the inflammation/edema/hypoxia cycle

Normal wound healing proceeds through an orchestrated sequence of hemostasis, inflammation, proliferation, and remodeling, driven by complex molecular and cellular interactions between the skin, vasculature, immune system, and extracellular matrix (ECM). In chronic wounds, these processes are often disrupted by the interrelated and self-sustaining effects of tissue hypoxia, persistent inflammation, lymphatic dysfunction, and edema.

### Wound bed hypoxia

Wounds are inherently hypoxic, as the metabolic demands of healing tissue often exceed the available oxygen supply.^3^ The initial injury disrupts local microvasculature, immediately impairing oxygen delivery. As inflammation ensues, metabolic demand increases due to the oxygen-consumptive generation of reactive oxygen species (ROS) for microbial defense and debris clearance. Concurrently, inflammatory cytokines drive vascular permeability, leading to fluid and leukocyte accumulation in the interstitial space (edema). Edema compresses capillaries and increases the diffusion distance for oxygen, compounding tissue hypoxia.^4^

### Role of lymphatics in inflammation

Edema is both a symptom and a driver of lymphatic disfunction. Adequate lymphatic function is essential for clearing inflammatory mediators, resolving inflammation, and maintaining tissue homeostasis.^5^ In wound healing, lymphatic clearance represents a critical, rate-limiting step, necessary to transition from inflammation to tissue repair.^6^ However, excessive edema, immobility, prolonged inflammation, and tissue fibrosis all significantly impair lymphatic function.^4^ Lymphatic overload results in inadequate clearance of inflammatory mediators, persistent edema, decreased tissue perfusion, increasingly amplified recruitment and activation of immune cells, downregulation of lymphangiogenic factors such as vascular endothelial growth factor (VEGF)-C and VEGF-D, elevated metabolic demand, and progressively worsening tissue hypoxia.^4^ Without adequate lymphatic clearance, inflammation cannot resolve, and wound repair cannot progress.^6^, ^7^, ^8^ Edema management is the primary method for supporting lymphatic function in dermal tissue and is, therefore, essential to advance healing, regardless of wound etiology—although careful clinical oversight is necessary in patients with significant large or small vessel disease.^9^

### The inflammatory amplification feedback loop

Persistent inflammation drives ongoing recruitment of neutrophils and macrophages, sustained release of pro-inflammatory cytokines, such as tumor necrosis factor-alpha (TNF-α), interleukin-1 beta (IL-1β) and interleukin-6 (IL-6), and continuous generation of ROS, reactive nitrogen species (RNS), and matrix metalloproteinases (MMPs), all of which perpetuate local tissue injury.^10^ The inflammatory microenvironment inhibits the transition of macrophages from pro-inflammatory M1 phenotypes to reparative M2 phenotypes, consequently reducing production of anti-inflammatory cytokines, such as interleukin-10 (IL-10), and specialized pro-resolving mediators (SPMs) essential for efferocytosis (the process by which phagocytic cells, such as macrophages, engulf and clear apoptotic cells).^11^^,^^12^

**Fig 1:**
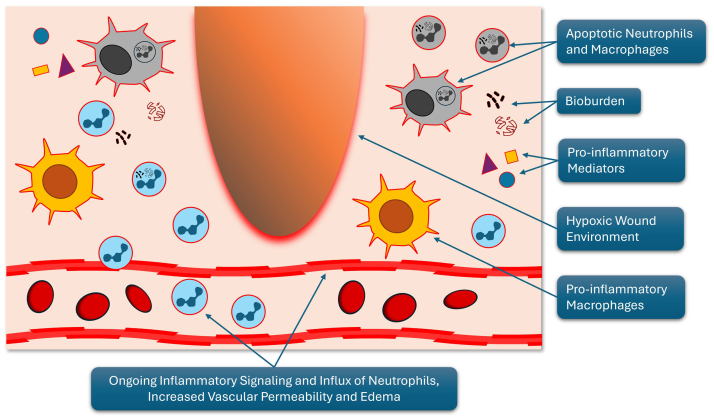
Inflammatory wound environment. The chronic inflammatory state of a nonhealing wound, characterized by ongoing inflammatory signaling, increased vascular permeability, persistent edema, and the accumulation of proinflammatory mediators, neutrophils, and proinflammatory macrophages. Additional features include bioburden, hypoxic tissue, and apoptotic immune cells. Together, these factors sustain inflammation and impair healing. (Illustration by MM Blakely, 2024. Presented by JM Lhor at: VEITH Symposium, November 21, 2024, New York, NY).

**Fig 2:**
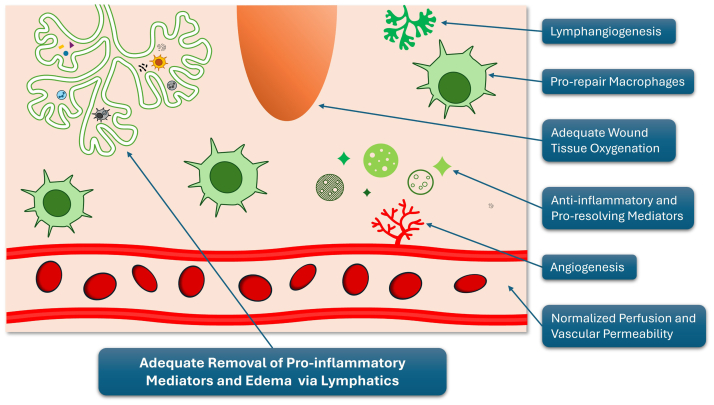
Resolution of inflammation. The wound microenvironment after successful resolution of inflammation. Hallmarks include effective lymphatic clearance of proinflammatory mediators and edema; a shift toward prorepair macrophages; increased lymphatic angiogenesis and angiogenesis; improved tissue oxygenation; and normalized perfusion and vascular permeability. These changes create a supportive environment for tissue repair and wound healing. (Illustration by MM Blakely, 2024. Presented by JM Lhor at: VEITH Symposium, November 21, 2024, New York, NY).

Inflamed, hypoxic wound environments promote microbial growth and biofilm formation, further exacerbating immune activation and metabolic demand, and increasing the risk of recurrent infection, osteomyelitis, progressive tissue necrosis, and systemic infection.^13^^,^^14^ Collectively, these processes inhibit inflammation resolution, accelerate ECM degradation and fibrosis, worsen lymphatic dysfunction, and reinforce the self-sustaining cycle of inflammation, edema, hypoxia, and tissue injury.^15^^,^^16^ The inflammatory wound environment is shown in Fig 1, and the wound environment after successful resolution of inflammation is shown in Fig 2.^17^

### Systemic implications

Importantly, the consequences of impaired lymphatic clearance and sustained inflammation extend beyond localized wound pathology. This pathological process has been linked to numerous chronic inflammatory diseases, such as cardiovascular disease, autoimmune conditions, neurodegenerative disorders such as Alzheimer’s, cirrhosis, hypertension, and renal dysfunction.^18^^,^^19^

### Ischemia/reperfusion

Further compounding the cycle of hypoxia, inflammation, and lymphatic dysfunction, lower extremity wounds are particularly vulnerable to ischemia/reperfusion (I/R) injury. In the upright position, gravitational forces increase hydrostatic pressure in the lower limbs, which promotes interstitial fluid accumulation and further impairs microvascular perfusion and tissue oxygenation. Lymphatic vessels, already impaired by inflammation, are unable to keep up with this additional workload. As a result, edema intensifies, inflammatory signaling escalates, and tissue hypoxia worsens.^20^

When the legs are elevated, such as when resting or sleeping, reduced hydrostatic pressure temporarily restores perfusion and reoxygenates ischemic tissues. Abrupt reoxygenation, however, triggers a burst of ROS and RNS that activate MMPs, amplify proinflammatory cytokines, and cause oxidative injury to the microvascular endothelium.^20^ In proinflammatory environments, particularly during I/R injury, excess nitric oxide (NO) and superoxide (O_2_^-^) combine to form peroxynitrite (ONOO^−^), a potent oxidizing and nitrating species. Peroxynitrite inflicts widespread cellular injury by damaging mitochondria, DNA, lipids, and proteins, leading to post-translational modifications such as tyrosine nitration, enzyme inactivation, and loss of cellular function.^10^^,^^21^ Clinical studies of chronic leg ulcer tissue have demonstrated elevated markers of peroxynitrite activity, including nitrotyrosine residues, and increased activation of the DNA damage sensor enzyme PARP-1, consistent with ongoing nitrosative stress.^22^ Although physiological levels of NO are vasoprotective and pro-angiogenic, its pathological overproduction in the setting of oxidative stress shifts signaling toward peroxynitrite formation. This imbalance is a major driver of endothelial dysfunction and has been implicated not only in impaired wound healing but also across a spectrum of chronic inflammatory diseases.^10^

Repeated episodes of I/R progressively dysregulate the inflammatory response. Cumulative oxidative and nitrosative stress and ongoing leukocyte infiltration damage the microvascular endothelium, promote fibrotic tissue remodeling, and impair vasoreactivity. Ultimately, these effects perpetuate a cycle of chronic vascular dysfunction, persistent inflammation, and delayed wound repair.^14^^,^^20^^,^^23^ Pressure injuries exhibit a similar I/R pattern, where sustained external loading induces local ischemia and subsequent off-loading produces reperfusion-mediated oxidative injury.^23^ Preventing I/R episodes through consistent edema management and protection from occlusive pressure is critical to inflammation resolution and wound healing.

### Contributing comorbidities

Comorbidities such as obesity, arterial insufficiency, venous insufficiency, diabetes mellitus, sedentary lifestyle, and nutritional deficiencies exacerbate abnormalities in tissue perfusion, inflammatory regulation, endothelial activity, oxidative stress, proteolysis, and tissue repair.^24^

#### Obesity

Obesity is associated with chronic low-grade inflammation and impaired lymphatic function. Adipose tissue secretes proinflammatory cytokines such as TNF-α and IL-6, which promote tissue edema, oxidative stress, lymphatic dysfunction, tissue fibrosis, and delayed healing.^4^

#### Arterial disease

Arterial disease worsens tissue hypoxia and limits regenerative capacity by reducing microvascular perfusion, impairing angiogenesis, and diminishing tissue responsiveness to growth factors like VEGF and platelet-derived growth factor (PDGF).^25^

#### Venous insufficiency

Venous insufficiency exacerbates many of the pathological processes underlying chronic wounds, particularly by intensifying I/R injury. Elevated venous pressure disrupts normal capillary pressure gradients, increasing resistance to arterial inflow and limiting oxygen delivery to tissues. Endothelial dysfunction and increased vascular permeability allow excess fluid and large inflammatory proteins to accumulate in the interstitium, resulting in persistent edema, decreased perfusion, prolonged inflammation, lymphatic dysfunction, and progressive tissue fibrosis.^20^^,^^23^ Venous leg ulcers are particularly susceptible to delayed healing in the presence of combined arterial and venous insufficiency, which is present in 15% to 20% of the venous leg ulcer population.^26^

#### Diabetes

Diabetes mellitus disrupts almost every aspect of wound healing. Chronic hyperglycemia elevates oxidative stress, impairs leukocyte chemotaxis and phagocytosis, and promotes formation of advanced glycation end-products (AGEs) that stiffen the ECM and damage nerve, vascular, and connective tissues.^25^^,^^27^ Inflammatory thickening and decreased elasticity of connective tissue and vessel walls leads to reduced joint mobility and vascular disease. Changes in foot architecture and loss of protective sensation increase the risk of repeated mechanical trauma and amplified inflammatory signaling. Autonomic neuropathy leads to dysregulation of the vasomotor response and, even in those with preserved arterial flow, altered capillary pressure gradients cause blood flow to bypass microvascular capillaries, decreasing tissue perfusion and increasing venous hypertension and edema.^27^^,^^28^

#### Other systemic comorbidities

**Fig 3:**
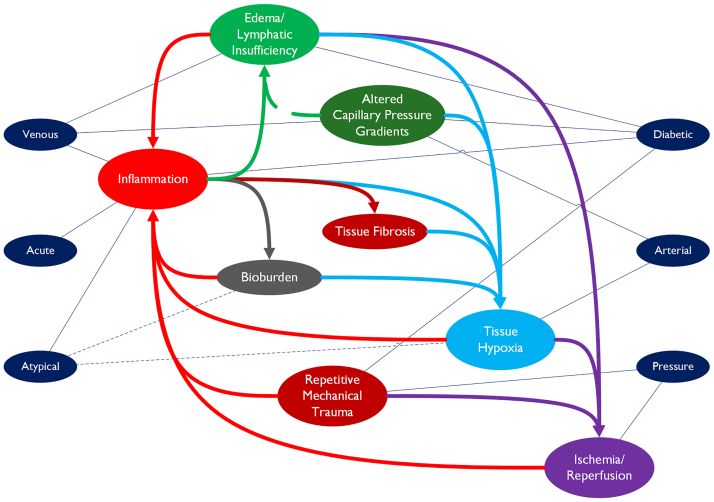
The inflammation/edema/hypoxia cycle. The interconnected mechanisms driving chronic wound pathology. Wound types (*navy nodes*) connect to primary causative factors. *Arrows* indicate downstream effects that lead to self-sustaining feedback loops involving inflammation, edema/lymphatic insufficiency, altered capillary pressure gradients, tissue hypoxia, ischemia-reperfusion (I/R) injury, tissue fibrosis, repetitive mechanical trauma, and bioburden. (Illustration by MM Blakely, 2025).

Other comorbidities, such as immobility, renal disease, cardiovascular conditions, autoimmune disorders, pulmonary disease, advanced age, and malnutrition, compound these effects by compromising systemic homeostasis and limiting the cellular energy, protein synthesis, perfusion, and immune function necessary for coordinated tissue repair.^29^^,^^30^ The interplay of factors involved in chronic wounds is shown in Fig 3.

## Key Mechanisms of Wound Healing

### The essential role of oxygen

Oxygen is essential in all phases of wound healing, supporting immune responses, collagen synthesis, angiogenesis, and the regeneration of new tissue. Adequate tissue oxygenation is critical for neutrophil, macrophage, and lymphocyte function, as well as for the generation of ROS and NO, which play key antimicrobial and regulatory roles throughout the healing process.^31^

#### Oxygen-dependent immune defense

During the initial inflammatory response, neutrophils and macrophages use available molecular oxygen to generate ROS via nicotinamide adenine dinucleotide phosphate (NADPH) oxidase in a process called the oxidative burst. O_2_^-^ is formed and then rapidly converted to hydrogen peroxide (H_2_O_2_) and hypochlorous acid (HOCl), potent antimicrobial agents that clear pathogens and debris. Concurrently, oxygen availability facilitates NO synthesis from L-arginine via NOS, supporting vasodilation, leukocyte migration, and immune signaling.^31^

#### Redox signaling

At appropriate levels, ROS serve as critical signaling molecules, a process known as redox signaling. Redox signaling regulates key aspects of wound healing, including immune modulation, angiogenesis, cellular proliferation and migration, and ECM remodeling.^31^ Through activation of transcription factors such as nuclear factor kappa B (NF-κB), hypoxia-inducible factor-1 alpha (HIF-1α), and activator protein-1 (AP-1), redox signaling coordinates immune responses, growth factor synthesis, and tissue repair.^31^ As inflammation transitions toward resolution, redox-sensitive pathways promote the biosynthesis of SPMs, which are essential for resolving inflammation and initiating tissue repair.^16^ In the proliferative phase, redox signaling facilitates endothelial cell activation, fibroblast proliferation, and keratinocyte migration, supporting angiogenesis, granulation tissue formation, and wound closure.^32^^,^^33^

A tightly regulated redox balance is essential for wound healing. Insufficient ROS can hinder antimicrobial defense and angiogenesis, whereas excess ROS cause oxidative stress, ECM degradation, and cellular senescence. Chronic wounds are often characterized by dysregulated redox signaling, with persistent oxidative stress disrupting the healing process.^31^

#### Nitric oxide

NO is synthesized from L-arginine by NOS, a process that requires molecular oxygen as a substrate. In the inflammatory phase, NO supports immune defense by mediating the oxidative burst in neutrophils and macrophages, providing direct antimicrobial activity and contributing to biofilm disruption.^34^ Moreover, physiologic NO signaling moderates excessive inflammation, facilitates macrophage polarization toward reparative M2 phenotypes, and fosters the biosynthesis of SPMs, which drive resolution of inflammation.^35^^,^^36^

As a potent vasodilator, NO regulates vascular tone, enhances blood flow, and improves oxygen delivery to ischemic tissues.^37^ NO plays a central role in angiogenesis by stimulating endothelial proliferation, migration, and microtubular formation, while interacting with growth factors such as VEGF and PDGF to promote angiogenesis and lymphatic angiogenesis.^38^ Beyond its vascular and immune functions, NO enhances fibroblast proliferation, collagen synthesis, and crosslinking, and promotes keratinocyte migration and proliferation, thus improving tissue tensile strength and accelerating re-epithelialization and wound closure.^34^

Dysregulated NO production can be detrimental, as insufficient levels impair perfusion, angiogenesis, and immune defense, while excessive production—particularly via inducible nitric oxide synthase (iNOS) in proinflammatory environments—leads to peroxynitrite formation, endothelial dysfunction, and oxidative/nitrosative injury.^10^

#### Tissue oxygen tension and metabolic efficiency in wound healing

**Fig 4:**
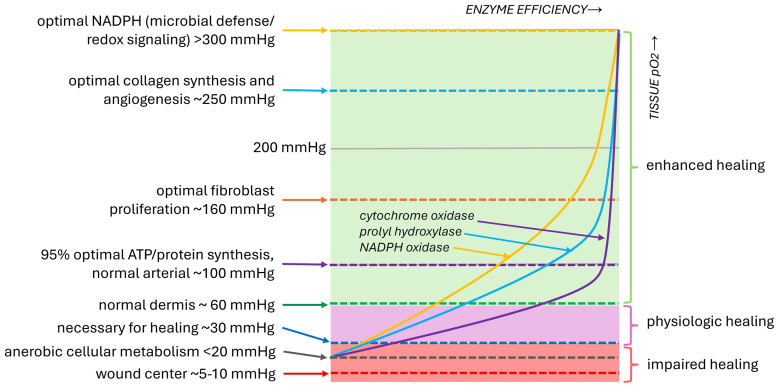
Enzymatic activity by oxygen tension. Wound repair is impaired below 30 mmHg partial pressure of oxygen (*pO*_*2*_), with a critical threshold at <20 mmHg where cells switch to anaerobic metabolism and oxygen-dependent processes fail; oxygen levels at wound centers commonly drop below 10 mmHg. Physiologic healing occurs between 30 and 60 mmHg, with ∼60 mmHg representing normal dermal pO_2_, while supplemental oxygen above 60 mmHg enhances enzymatic activity, immune defense, collagen synthesis, and angiogenesis. Distinct enzyme curves illustrate the nonlinear dependence of healing pathways on oxygen availability. *ATP*, Adenosine triphosphate; *NADPH*, nicotinamide adenine dinucleotide phosphate. (Illustration by MM Blakely, 2025).

Adequate tissue oxygen tension (pO_2_) is critical for optimal wound healing. Normal arterial tissue pO_2_ is approximately 100 mmHg, but even at this level, key oxygen-dependent processes function below their maximal efficiency. For example, at 100 mmHg, microbial defense mechanisms operate at ∼60% capacity, collagen synthesis at ∼80%, and ATP production—essential for protein synthesis, cell proliferation, and repair—at approximately 95% efficiency (Fig 4).^44^, ^45^

Well-vascularized dermal tissue has an oxygen tension of 40 to 80 mmHg.^41^, ^42^ In chronic wounds, oxygen tension frequently falls below 10 mmHg.^31^, ^42^ Tissue oxygen tensions below 30 mmHg are generally insufficient for effective wound healing, limiting inflammation resolution, fibroblast function, collagen synthesis, and angiogenesis.^31^, ^43^ Given that physiological oxygen-dependent mechanisms remain submaximal even at normal tissue pO2 levels, supplemental oxygen therapy may enhance healing beyond that achievable under normal conditions. Therefore, therapies aimed at increasing local tissue oxygenation present considerable therapeutic potential for chronic wound healing and limb preservation.

### Endothelial cell activation

Endothelial cells (ECs) are specialized cells that line the interior surface of blood and lymphatic vessels and serve as dynamic regulators of vascular function, immune response, and tissue repair.^37^ ECs respond dynamically to shear stress and oxygen levels to coordinate inflammatory resolution, angiogenesis, and tissue repair.^38^

ECs are highly sensitive to mechanical forces, which they translate into biochemical signals through a process called mechanotransduction.^44^^,^^45^ The primary mechanosensor of ECs is the glycocalyx (eGC), a negatively charged, proteoglycan-rich network that lines the luminal surface. The eGC stabilizes vascular permeability, provides anticoagulant and anti-adhesive properties, and transduces shear stress into biochemical mediators, such as NO, and prostacyclin, which support vascular homeostasis, and redox balance.^46^ When compromised by inflammatory cytokines, ROS, and RNS, the eGC becomes degraded, resulting in increased permeability, leukocyte adhesion, and dysregulated vasodilation.^46^ Destruction and impairment of the eGC promotes edema, inflammation, and vascular dysfunction—features commonly observed in diabetes, venous insufficiency, and arterial disease.^21^ In contrast, laminar shear stress from healthy flow stabilizes the eGC and maintains vascular homeostasis.^46^

In wound repair, hypoxia, inflammatory signaling, and biomechanical forces activate ECs, which—together with fibroblasts and macrophages—release growth factors including VEGF, PDGF, and fibroblast growth factor (FGF). The established chemoattractant gradients recruit ECs into the wound bed, where they proliferate and organize into new vessels.^47^^,^^48^ Beyond angiogenesis, ECs regulate ECM remodeling by releasing MMPs and tissue inhibitors of metalloproteinases (TIMPs), modulating fibroblast activity, coordinating granulation tissue formation, and promoting re-epithelialization.^32^^,^^49^ Lymphatic endothelial cells (LECs) are similarly activated and play critical roles in homeostasis by regulating interstitial fluid balance, clearance of inflammatory mediators and immune cell trafficking. LEC dysregulation impairs lymphatic angiogenesis and contributes to persistent edema, inflammatory cytokine accumulation, delayed healing, and tissue fibrosis.^6^^,^^11^

### Specialized pro-resolving lipid mediators

SPMs—including lipoxins, resolvins, protectins, and maresins—are bioactive molecules derived from omega-3 and omega-6 polyunsaturated fatty acids, synthesized locally at sites of inflammation. SPMs act as key orchestrators of antimicrobial defense, inflammation resolution, tissue repair, and pain modulation. Guided by inflammatory signaling, tissue oxygen tension, and biomechanical forces, SPMs exert overlapping, complementary effects throughout the sequential phases of wound repair.^12^^,^^50^

#### Inflammatory phase

In the earliest stages of injury, lipoxins limit excessive neutrophil infiltration, suppress proinflammatory cytokine release, and promote monocyte recruitment, thereby reducing the intensity and duration of inflammation.^51^ Resolvins enhance microbial defense, disrupt biofilms, and initiate efferocytosis, collectively reducing oxidative stress and limiting collateral tissue injury.^52^^,^^53^

#### Resolution and transition to repair

As inflammation subsides, resolvins drive macrophage polarization toward the reparative M2 phenotype and reinforce efferocytosis, supporting lymphatic clearance of apoptotic cells, microbes, and debris.^11^^,^^54^ Lipoxins amplify IL-10 and TGF-β signaling, reinforcing anti-inflammatory pathways and priming fibroblasts for repair.^55^^,^^56^

#### Proliferative phase

During tissue repair, resolvins stimulate endothelial activation and VEGF expression, promoting angiogenesis and lymphatic angiogenesis to restore tissue perfusion and support clearance of inflammatory mediators.^50^ Protectins shield endothelial cells from oxidative injury and support fibroblast proliferation, granulation tissue formation, keratinocyte migration, and re-epithelialization.^12^ Maresins, synthesized by macrophages during this stage, promote ECM synthesis, collagen deposition, myofibroblast differentiation, and wound contraction.^12^

#### Remodeling and maturation phase

In later stages, maresins and M2 macrophages modulate TGF-β signaling to regulate collagen alignment, tensile strength, and tissue remodeling. The tissue remodeling phase reduces fibrosis and scarring while encouraging the development of structurally organized, functionally durable tissue.^12^^,^^57^

#### Pain modulation

Lipoxins, resolvins, and protectins reduce pain throughout the wound-healing process by suppressing inflammatory signaling, enhancing efferocytosis, and upregulating tissue-protective mediators.^12^ Resolvins and maresins provide both anti-inflammatory and neuroprotective pain relief by directly modulating nociceptive pathways, including opioid receptors and transient receptor potential (TRP) channels.^58^

#### Oxygen dependence of SPM biosynthesis

Importantly, SPM biosynthesis is dependent on local oxygen availability. Multiple enzymes involved in their generation, including 15-lipoxygenase (15-LOX) and 5-lipoxygenase (5-LOX), require molecular oxygen as a substrate.^12^^,^^54^ The aerobic nature of SPM synthesis directly links tissue oxygen tension to the efficiency of inflammation resolution and tissue repair.

### Autophagy

Autophagy is a critical cellular mechanism for defending against unregulated oxidation and inflammation. It is a complex lysosomal catabolic process by which cells degrade or recycle their contents to maintain cellular homeostasis, adapt to stress, and respond to disease.^59^ Autophagy and SPMs are mutually reinforcing resolution pathways, where autophagy enhances the biosynthetic machinery for SPM production, while SPMs in turn optimize autophagy to clear debris, suppress excessive inflammation, and protect tissues.^59^ In tissue repair, autophagy facilitates keratinocyte migration, fibroblast proliferation, and angiogenesis by recycling intracellular components to fuel biosynthesis and adenosine triphosphate (ATP) production.^60^^,^^61^

## Therapeutic approaches to address hypoxia, inflammation, and lymphatic dysfunction

Given the pivotal roles of tissue hypoxia, sustained inflammation, impaired lymphatic drainage, and persistent edema in chronic wound pathogenesis, therapeutic approaches that specifically address these issues are of considerable clinical value. Two noninvasive modalities, topical oxygen therapy (TOT) and intermittent compression (IC), have emerged as promising, mechanism-based interventions.

### Topical oxygen therapy

TOT delivers concentrated oxygen directly to the wound bed, bypassing systemic perfusion limitations and raising local tissue oxygen tension.^32^ By increasing local tissue oxygen tension, TOT supports crucial oxygen-dependent processes, including microbial defense via oxidative burst, collagen synthesis, angiogenesis, and cellular proliferation.^3^^,^^42^

Mechanistic studies provide compelling evidence of TOT’s biological efficacy. Fries et al demonstrated in a porcine model that topical oxygen significantly increased tissue oxygen tension from a hypoxic baseline of 11 mmHg to over 40 mmHg within 4 minutes, penetrating 2 mm into the wound tissue, where the oxygen sensor was placed. Elevated oxygen levels persisted at therapeutic concentrations for 15 days after cessation of a 7-day treatment regimen due to increased localized angiogenesis and new capillary formation. Histologically, TOT-treated wounds showed significantly increased VEGF expression, enhanced angiogenesis, increased fibroblast proliferation, and more organized collagen fiber deposition. The cellular-level changes correlated clinically with improved granulation tissue quality, reduced necrosis, accelerated epithelialization, and faster wound contraction compared with controls.^32^ Gottrup et al observed similar O_2_ diffusion depth, as well as accelerated epithelialization, and improved granulation tissue formation in TOT-treated wounds.^62^

Biofilm formation is frequently associated with recurrent wound infection and delayed healing, due to decreased metabolic activity, limiting antimicrobial effectiveness, and low oxygen tension, inhibiting oxidative burst.^13^^,^^14^ Ball et al demonstrated in a porcine model that TOT increased biofilm metabolic activity, thereby increasing antimicrobial susceptibility.^63^ Tawfick et al found reductions in methicillin-resistant *Staphylococcus aureus* (MRSA)-positive wounds pre vs post TOT treatment.^64^ Song et al compared TOT + negative pressure wound therapy (NPWT) with NPWT alone in 112 chronic traumatic wounds and found significantly reduced bacterial culture-positive rates in the TOT + NPWT group compared with baseline and in the TOT + NPWT group compared with the NPWT alone group. They also found larger increases in tissue oxygen tension, greater reductions in pain scores, and accelerated granulation tissue formation in the TOT group.^65^

Additional mechanistic support comes from in vitro studies. Gordillo and Sen found exposure of human dermal fibroblasts to hyperoxic conditions increased transforming growth factor-beta 1 (TGF-β1) expression, stimulated collagen production, and activated redox-sensitive transcription factors such as HIF-1α and AP-1, linking oxygen tension to genes involved in matrix remodeling.^66^ Heng et al reported increased capillary density, and enhanced neutrophil activity and fibroblast proliferation in TOT-treated wounds.^67^ In an oral mucosa human study, TOT compared with control increased wound healing for gingival grafts, with histomorphometric evidence of increased vessel area, number and caliber of vessels, and a higher microvessel density.^68^ Tawfick and Sultan showed increased VEGF expression and endothelial proliferation in tissue biopsies from TOT-treated wounds.^64^

Clinical studies further support the molecular and basic science findings. Lavery et al, in a prospective study of patients with diabetic foot ulcers (DFUs), found TOT significantly increased tissue oxygenation and elevated levels of cytokines critical to healing, including TGF-β, VEGF, TNF-α, and IL-6. Clinically, more than one-half of the treated patients achieved at least a 50% reduction in wound size within 3 weeks.^69^ Dissemond et al observed that TOT improved pain, infection control, and wound closure, and proposed TOT enhanced neutrophil oxidative burst and promoted macrophage polarization toward the pro-repair M2 phenotype.^70^

Additional insights from Sen and Headland and Norling emphasize the role of pO_2_ in macrophage polarization and the oxygen-dependent biosynthesis of SPMs.^31^^,^^55^ Collectively, mechanistic and clinical findings demonstrate that TOT not only compensates for the inherent hypoxic conditions of chronic wounds but actively modulates the wound microenvironment, enhancing immune regulation, vascular and matrix remodeling, and overall tissue regeneration.

### Intermittent compression

IC therapy provides therapeutic benefits through synergistic effects involving the macrovascular and cellular mechanisms. By cyclically applying external mechanical pressure to the affected limb, IC enhances venous and lymphatic drainage, reduces edema, and restores interstitial fluid homeostasis.^71^ The macrovascular effects directly decrease oxygen diffusion distances within tissues, normalize capillary pressure gradients, and subsequently enhance local tissue perfusion and oxygen delivery.^71^ Additionally, the reduction of venous pressure achieved through IC indirectly improves arterial inflow by lowering arteriolar resistance, making this intervention safe and beneficial even in patients with compromised arterial circulation.^9^^,^^72^^,^^73^

At a cellular level, IC delivers both shear stress (tangential fluid forces) and shear stretch (mechanical deformation), which mechanosensitive cells, such as endothelial cells, macrophages, fibroblasts, and keratinocytes, translate into intracellular signaling cascades, modulating inflammation, immune function, and tissue repair.^74^^,^^75^

Endothelial cells respond to shear stress by releasing NO, VEGF, and anti-inflammatory mediators, promoting angiogenesis, vasodilation, and optimized immune cell trafficking.^76^ In the lymphatic vasculature, mechanical forces drive nearly all aspects of development and function.^77^ A cytoskeletal filament network, comprised of actin filaments, intermediate filaments, and microtubules, anchors lymphatic endothelial cells to the ECM, making the structural network highly responsive to interstitial pressure, tissue stretch, and fluid shear stress.^4^ The cytoskeletal structural network functions as both a scaffold, providing stability under fluctuating tissue forces, and a signaling modulus, translating mechanical cues into biochemical responses. Mechanotransduction in LECs regulates lymphatic angiogenesis, vessel maturation, and contractile pumping, while coordinating immune trafficking and the production of mediators that resolve inflammation.^4^

Macrophages under mechanical stimulation shift toward a reparative M2 phenotype, synthesizing SPMs and reducing proinflammatory cytokine production.^74^ Fibroblasts demonstrate enhanced migration, proliferation, collagen and ECM synthesis, and differentiation into myofibroblasts, which support wound contraction and structural remodeling.^78^ Keratinocytes display improved migration and proliferation, accelerating re-epithelialization. Additionally, mechanical stimulation promotes orderly collagen alignment, limiting fibrosis and scar formation.^74^

Across in vitro, animal, and clinical studies, IC consistently demonstrates pro-healing vascular and cellular effects with meaningful patient outcomes. In endothelial models, simulated external limb compression increased NO bioavailability and endothelial nitric oxide synthase (eNOS) expression (≈2-fold within 6 hours) in human umbilical vein ECs,^79^ whereas leg IC in vivo increased eNOS expression by 1.8-fold in upstream muscle.^80^ Additional work demonstrated upregulation of VEGF and monocyte chemotactic protein-1 (MCP-1) messenger RNA (mRNA) expression following 150 minutes of IC in rat limbs.^81^

In vivo and clinical investigations confirm these vascular effects and demonstrate that IC improves perfusion and endothelial function, reduces edema, and enhances functional capacity.^71^^,^^82^^,^^83^ In acute ankle fracture management, pedal IC significantly reduced edema within 24 to 48 hours.^84^ In patients with lower-limb edema and impaired mobility, IC reduced swelling, improved ankle range of motion, and improved quality of life.^85^ Gastrointestinal surgery patients treated with IC demonstrated increased plantar perfusion, measured by plantar deep temperature.^86^

In connective tissue healing models, IC enhanced fibroblast density (+53%), vessel density (+64%), sensory neuropeptides (substance P +110%, calcitonine gene-related peptide +47%), and collagen fiber organization, supporting improved neurovascular ingrowth and tissue repair.^87^

IC has also been extensively evaluated in peripheral arterial disease. In patients with intermittent claudication, home IC increased walking distance by >100%, improved resting ankle/brachial index (ABI) (+18%), post-exercise ABI (+110%), and arterial calf inflow (+36%) after 4.5 months, with benefits sustained for 1 year.^88^ Later work confirmed greater improvements when both foot and calf were treated vs either region alone.^73^ De Haro et al showed that 3 months of IC significantly increased initial claudication distance (+66%), absolute claudication distance (+52%), and post-exercise ABI (+42%), with durable benefit at follow-up.^89^

In patients with critical limb ischemia and nonhealing foot wounds, where revascularization was no longer an option, IC achieved complete wound healing and limb salvage in 58% compared with 17% of controls, while amputation rates were significantly lower in the IC group compared with the control group.^90^

Collectively, these data indicate that IC favorably modulates endothelial biology and microcirculatory dynamics, accelerates edema resolution, and translates into improved clinical, functional, and limb salvage outcomes.

## An integrated approach: multimodality intermittent topical oxygen therapy

**Fig 5:**
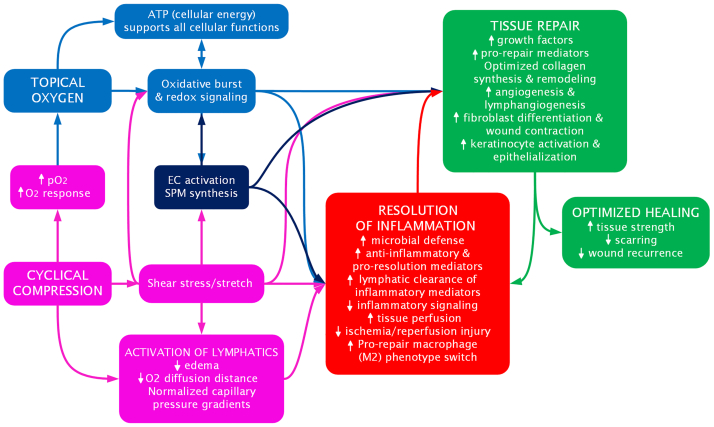
Multi-modality intermittent toical oxygen therapy (ITOT) mechanism of action. The integrated cellular and molecular mechanisms by which topical oxygen and cyclical compression synergistically promote wound healing. Topical oxygen increases tissue oxygen tension to fuel adenosine triphosphate (*ATP*) production, enhance microbial defense via oxidative burst, activate redox signaling, and optimize collagen synthesis and crosslinking. Cyclical compression increases the partial pressure (*pO*_*2*_) of topical oxygen and activates lymphatic function, improving clearance of inflammatory mediators, reducing edema, decreasing diffusion distance for oxygen, normalizing capillary pressure gradients, and restoring perfusion. Compression-induced shear stress and shear stretch activate endothelial cells (*ECs*) and stimulate the biosynthesis of specialized pro-resolving mediators (*SPMs*). This initiates a cascade of anti-inflammatory and pro-resolution signaling, including polarization of macrophages toward the reparative pro-repair macrophage (*M2*) phenotype and acceleration of inflammation resolution. In parallel, M2 macrophages, SPMs, and ECs upregulate growth factors and reparative cytokines that direct wound repair and remodeling. Activated ECs stimulate angiogenesis and lymphatic angiogenesis, while fibroblasts drive collagen synthesis, ECM production, and myofibroblast differentiation, enabling wound contraction. Keratinocyte activation promotes epithelialization, and during remodeling, fibroblast activity enhances collagen fiber organization. Collectively, these pathways promote efficient resolution of inflammation, improved perfusion, increased tissue strength, reduced scarring, and lower wound recurrence. *NADPH*, Nicotinamide adenine dinucleotide phosphate. (Illustration by Blakely MM, 2025).

Multi-modality intermittent topical oxygen therapy (ITOT) combines noncontact cyclical compression with pressurized topical oxygen delivery in a single device to simultaneously address hypoxia, edema, and inflammation (optional humidification is available with the device, to counteract any oxygen-related wound desiccation). By leveraging complementary mechanisms, ITOT potentiates the therapeutic benefits of each modality and optimizes wound healing through multiple, synergistic pathways. The key mechanisms of action and synergistic effects of combined therapy are shown in the Table and Fig 5.

**Table:** Multi-modality intermittent topical oxygen therapy (*ITOT*) mechanism of action

Clinical/cellular outcome	TOT^12^^,^^31^^,^^32^^,^^47^^,^^50^^,^^39^^,^^98^, ^99^, ^100^	Cyclical compression^4^^,^^11^^,^^50^^,^^71^, ^72^, ^73^^,^^82^^,^^83^^,^^101^, ^102^, ^103^, ^91^	Combined therapy (ITOT)^47^^,^^48^^,^^50^^,^^64^^,^^65^, ^74^^,^^98^^,^^99^, ^93^
Microbial defense	↑ ROS generation ↑ SPM microbial clearance	↑ Vascular/lymphatic immune cell trafficking ↑ ROS generation ↑ SPM microbial clearance	↑ Immune cell access ↑ ROS-driven killing ↑ Biofilm disruption ↑ Lymphatic microbial clearance
Pain modulation and protective pathways	SPMs inhibit nociceptive signaling and activate reparative programs	↑ Perfusion SPM tissue protection ↓ I/R injury ↓ Pain	↓ Pain and oxidative tissue damage
Resolution of inflammation	Redox signaling & SPMs: ↓ Inflammatory cytokines ↑ M2 macrophage polarization	↑ Lymphatic removal of inflammatory mediators ↑ SPM pro-resolution mediators ↑ Anti-inflammatory EC signaling	↑ SPM production ↑ Lymphatic clearance ↑ Immune modulation ↓ Collateral tissue damage
Tissue perfusion	↑ Local oxygen availability	↑ pO_2_ ↓ O_2_ diffusion distance ↑ Arterial inflow Normalized capillary pressure gradients	↑ Perfusion ↑ Oxygen penetration and efficacy ↓ I/R injury
Angiogenesis	↑ VEGF, PDGF, and NO production ↑ EC proliferation and tube formation	ECs activation normalizes capillary pressure gradients and promotes capillary sprouting	Synergistic mechanical and chemical EC activation and growth factor signaling
Tissue repair	↑ ATP production ↑ Fibroblast activation Optimal collagen synthesis	↑ ECM production and fibroblast alignment	Stronger, more functional granulation tissue and wound matrix
Epithelialization	↑ Keratinocyte migration and proliferation via ATP and redox balance	Cytoskeletal activation and directional migration of keratinocytes	Faster, more complete re-epithelialization via combined energy and mechanical signaling
Remodeling	↑ Collagen synthesis, crosslinking, and fibroblast remodeling	↑ Fibroblast migration and collagen fiber alignment	More mature and organized ECM ↑ Tissue strength ↓ Wound recurrence ↓ Scarring

*ATP,* Adenosine triphosphate; *EC,* endothelial cell; *ECM,* extracellular matrix; *I/R,* ischemia/reperfusion; *NO,* nitric oxide; *PDGF,* platelet-derived growth factor; *pO*_*2*_*,* partial pressure of oxygen; *ROS,* reactive oxygen species; *SPM,* specialized pro-resolving mediator; *TOT,* topical oxygen therapy; *VEGF,* vascular endothelial growth factor.

### ITOT published evidence

Published clinical outcomes of ITOT highlight the benefits of cyclical pressure and TOT multi-modality approach as an adjunct to current best practice standard wound care.

Frykberg et al, in a rigorous double-blinded, randomized, controlled trial (RCT) of ITOT in DFUs, demonstrated a 41.7% closure rate at 12 weeks vs 13.5% in the control group (*P* = .004), a 56% closure rate at 12 months post enrollment vs 27% in the control group (*P* = .013), and a 6.7% recurrence rate at 12 months vs 40% in the control group (*P* = .070).^99^

Yellin et al, in a well-designed 202-patient retrospective study of ITOT in the treatment of DFUs in veterans, showed an 82% reduction in hospitalizations (7.1% vs 40%; *P* < .0001) and a 73% reduction in amputations (8.6% vs 31.4%; *P* = .0007) compared with a matched cohort, both at 12 months. The authors concluded more durable healing and corresponding reductions in wound recurrence, hospitalizations, and amputations were achieved with ITOT treatment, representing significant health economic benefits.^100^

Tawfick, et al in a 132-patient prospective controlled study, compared ITOT with conventional compression dressings (CCDs) in the management of nonhealing venous leg ulcers (VLUs), present for more than 2 years (range, 2-43 years). They showed a healing rate of 76% vs 46% (*P* < .0001), a median time to closure of 57 vs 107 days (*P* < .0001), and a healed wound recurrence rate of 6% (3 of 51) vs 47% (14 of 30) at 36 months, for patients treated with ITOT and CCDs, respectively (*P* < .0001). The pain score threshold of ITOT-treated patients improved from 8 of 10 to 3 of 10 by treatment day 13. Additionally, 11 of the 24 MRSA-positive ulcers in the TWO2 therapy group were MRSA-negative, and none of the 19 MRSA-positive ulcers in the CCD group were MRSA-negative after 5 weeks of treatment (*P* <.001).^64^^,^^98^

Blackman et al found DFUs treated with ITOT healed faster (56 days vs 93 days) and healed more ulcers (82% vs 45%) than standard care (*P* = .04).^101^

Sano et al, in a 6-patient prospective study, demonstrated ITOT effectively increased TcPO_2_ values from below 30 mmHg to above 50 mmHg in the periwound tissue of all treated subjects.^102^

Kerr et al conducted a formal cost-effectiveness analysis of ITOT for DFUs using National Health Service costing norms in England. Their findings showed ITOT both increased quality-adjusted life years and reduced overall treatment costs by an estimated 16% over 2 years.^103^

### Summary of ITOT published findings

• Higher incidence of healing;
• Reduced time to healing;
• Reduced infection;
• Improved granulation tissue formation;
• Pain reduction;
• Improved quality-of-life scores;
• Reduced scarring;
• Lower recurrence rates;
• Decreased hospitalizations;
• Decreased amputations;

## Conclusion

Chronic wounds persist due to a self-perpetuating cycle of tissue hypoxia, edema, persistent inflammation, and lymphatic dysfunction, exacerbated by I/R injury, bioburden, tissue fibrosis, and comorbidities. Interventions that concurrently address multiple drivers of wound chronicity hold significant therapeutic potential. TOT increases tissue oxygen tension, enhances microbial defense, and promotes inflammation resolution and through redox signaling and SPM synthesis. During tissue repair, TOT supports angiogenesis and optimal collagen synthesis, crosslinking, and ECM remodeling, leading to stronger tissue tensile strength and more durable wound healing. Cyclical compression improves lymphatic clearance of inflammatory mediators, reduces edema, restores perfusion, mitigates I/R injury, and activates mechanotransductive pathways supporting inflammation resolution, angiogenesis, and tissue repair. The combination of TOT and cyclical compression increases the partial pressure of oxygen available to wound tissue and exerts synergistic effects across multiple wound repair mechanisms. This integrative approach provides a promising adjunctive treatment strategy to accelerate healing, enhance clinical outcomes, reduce complications, and achieve durable closure in difficult wounds of varied etiologies.

## Author Contributions

Conception and design: JL, JF, DD, MR, ME, KO, MB

Analysis and interpretation: Not applicable

Data collection: Not applicable

Writing the article: JL, JF, DD, MB

Critical revision of the article: JL, JF, DD, MR, ME, KO

Final approval of the article: JL, JF, DD, MR, ME, KO, MB

Statistical analysis: Not applicable

Obtained funding: Not applicable

Overall responsibility: JL

## Funding

None.

## Disclosures

J.L. and J.R. are employed by the Veterans Administration; however, the views expressed in this article are those of the authors and do not represent the views of the Department of Veterans Affairs or the United States government. M.B. is a clinical investigator for AOTI. Inc. The remaining authors report no conflicts.
